# 3-(3,5-Dimethyl-1*H*-pyrazol-1-yl)propanamide

**DOI:** 10.1107/S160053680903342X

**Published:** 2009-09-12

**Authors:** Jian-Feng Zhang, Feng Huang, Shu-Jiao Chen

**Affiliations:** a State Key Laboratory Base of Novel Functional Materials and Preparation Science, Faculty of Materials Science and Chemical Engineering, Ningbo University, Ningbo, Zhejiang 315211, People’s Republic of China

## Abstract

In the crystal of the title compound, C_8_H_13_N_3_O, mol­ecules are linked by inter­molecular N—H⋯N and N—H⋯O hydrogen bonds into a three-dimensional network. Additional stabilization is provided by weak inter­molecular C—H⋯O hydrogen bonds.

## Related literature

For the potential applications of hemilabile ligands containing substituted pyrazole groups, see: Pal *et al.* (2005 ▶); Shaw *et al.* (2004 ▶). For the design of various pyrazole ligands with special structural properties to fulfill the specific stereochemical requirement of a particular metal-binding site, see: Mukherjee (2000 ▶); Paul *et al.* (2004 ▶);
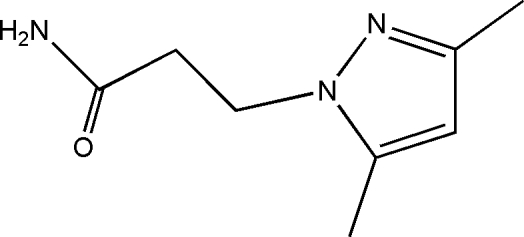


## Experimental

### 

#### Crystal data

C_8_H_13_N_3_O
*M* *_r_* = 167.21
Orthorhombic, 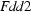

*a* = 14.452 (5) Å
*b* = 33.390 (7) Å
*c* = 7.4354 (15) Å
*V* = 3588.0 (16) Å^3^
*Z* = 16
Mo *K*α radiation
μ = 0.09 mm^−1^
*T* = 298 K
0.47 × 0.37 × 0.36 mm

#### Data collection

Rigaku R-AXIS RAPID diffractometer
Absorption correction: multi-scan (*ABSCOR*; Higashi, 1995 ▶) *T*_min_ = 0.963, *T*_max_ = 0.970
4623 measured reflections
1067 independent reflections
890 reflections with *I* > 2σ(*I*)
*R*_int_ = 0.044

#### Refinement

*R*[*F* ^2^ > 2σ(*F* ^2^)] = 0.033
*wR*(*F* ^2^) = 0.093
*S* = 1.14
1067 reflections
112 parameters
1 restraint
H-atom parameters constrained
Δρ_max_ = 0.14 e Å^−3^
Δρ_min_ = −0.19 e Å^−3^

### 

Data collection: *RAPID-AUTO* (Rigaku, 1998 ▶); cell refinement: *RAPID-AUTO*; data reduction: *CrystalStructure* (Rigaku/MSC, 2004 ▶); program(s) used to solve structure: *SHELXS97* (Sheldrick, 2008 ▶); program(s) used to refine structure: *SHELXL97* (Sheldrick, 2008 ▶); molecular graphics: *SHELXTL* (Sheldrick, 2008 ▶); software used to prepare material for publication: *SHELXL97*.

## Supplementary Material

Crystal structure: contains datablocks global, I. DOI: 10.1107/S160053680903342X/lh2847sup1.cif

Structure factors: contains datablocks I. DOI: 10.1107/S160053680903342X/lh2847Isup2.hkl

Additional supplementary materials:  crystallographic information; 3D view; checkCIF report

## Figures and Tables

**Table 1:** Hydrogen-bond geometry (Å, °)

*D*—H⋯*A*	*D*—H	H⋯*A*	*D*⋯*A*	*D*—H⋯*A*
N1—H1*A*⋯O1^i^	0.86	2.10	2.936 (3)	164
N1—H1*B*⋯N3^ii^	0.86	2.30	3.084 (3)	152
C3—H3*B*⋯O1^iii^	0.97	2.52	3.413 (3)	154

Symmetry codes: (i) 
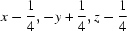
; (ii) 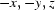; (iii) 
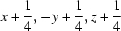
.

**Fig. 1.**
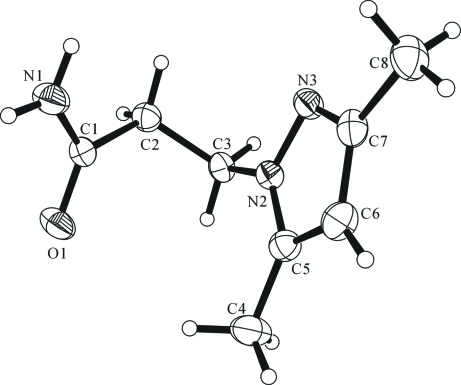
The molecular structure of the title compound. Displacement ellipsoids are drawn at the 30% probability level.

**Fig. 2.**
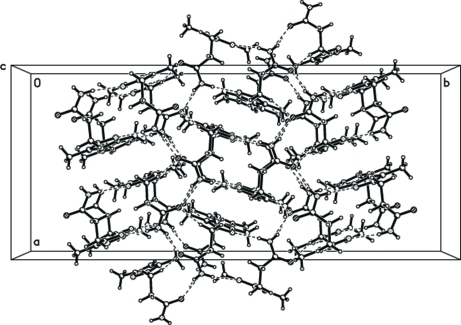
Part of the crystal structure of the title compound. Dashed lines indicate hydrogen bonds.

## supplementary crystallographic information

### Comment

In recent years, there has been considerable interest in the use of hemilabile ligands containing substituted pyrazole groups because of their potential applications in catalysis and their ability for complex construction (Shaw *et al.*, 2004; Pal *et al.*, 2005). Nowadays, much attention has been focused on the design of various pyrazole ligands with special structural properties to fulfill the specific stereochemical requirement of a particular metal-binding site (Mukherjee, 2000; Paul *et al.*, 2004). Herein, we report the crystal structure of the title compound. The molecluar structure of the title compound is shown in Fig. 1. In the crystal structure, molecules are linked by intermolecular N—H···N and N—H···O hydrogen bonds into a three dimensional network (see Fig. 2 and Table 1). Additional stabilization is provided by weak intermolecular C-H···O hydrogen bonds.

### Experimental

A mixture of 3,5-dimethylpyrazole (3.845 g, 40 mmol), sodium hydroxide (0.2 g, 5 mmol) and  *N*,*N*'-dimethylformamide(DMF)(100 ml) was stirred and heated to 373 K. A solution of acrylamide (2.843 g, 40 mmol) in DMF (20 ml) was added dropwise. After 6 h, heating was then terminated. The cooled reaction mixture was filtered and DMF was removed by vacuum distillation to give 3.66 g analytically pure *N*-pyrazolylpropanimide (yield: 54.7%). Recrystallization from ethanol solution yielded colorless single-crystals suitable for X-ray diffraction analysis. Calculated for C_8_H_13_N_3_O: C 57.42, H 7.78, N 25.12%; found: C 57.26, H 7.59, N 25.18%.

### Refinement

H atoms were positioned geometrically and treated in the subsequent refinement as riding atoms, with C—H = 0.93 (aromatic), 0.97 Å (methylene), 0.96 (methyl) and N—H = 0.86 Å, and with *U*_iso_(H) = 1.2 *U*_eq_(C,*N*) or 1.5 *U*_eq_(C_methyl_). In the absense of significant anomalous dispersion effects Friedel pairs were merged.

### Crystal data

**Table:**

C_8_H_13_N_3_O	*F*(000) = 1440
*M**_r_* = 167.21	*D*_x_ = 1.238 Mg m^−^^3^
Orthorhombic, *F**d**d*2	Mo *K*α radiation, λ = 0.71073 Å
Hall symbol: F 2 -2d	Cell parameters from 4623 reflections
*a* = 14.452 (5) Å	θ = 3.1–27.5°
*b* = 33.390 (7) Å	µ = 0.09 mm^−^^1^
*c* = 7.4354 (15) Å	*T* = 298 K
*V* = 3588.0 (16) Å^3^	Block, colorless
*Z* = 16	0.47 × 0.37 × 0.36 mm

### Data collection

**Table:**

Rigaku R-AXIS RAPID diffractometer	1067 independent reflections
Radiation source: fine-focus sealed tube	890 reflections with *I* > 2σ(*I*)
graphite	*R*_int_ = 0.044
Detector resolution: 0 pixels mm^-1^	θ_max_ = 27.5°, θ_min_ = 3.1°
ω scans	*h* = −17→18
Absorption correction: multi-scan (*ABSCOR*; Higashi, 1995)	*k* = −43→43
*T*_min_ = 0.963, *T*_max_ = 0.970	*l* = −9→8
4623 measured reflections	

### Refinement

**Table:**

Refinement on *F*^2^	Secondary atom site location: difference Fourier map
Least-squares matrix: full	Hydrogen site location: inferred from neighbouring sites
*R*[*F*^2^ > 2σ(*F*^2^)] = 0.033	H-atom parameters constrained
*wR*(*F*^2^) = 0.093	*w* = 1/[σ^2^(*F*_o_^2^) + (0.0446*P*)^2^ + 1.8694*P*] where *P* = (*F*_o_^2^ + 2*F*_c_^2^)/3
*S* = 1.14	(Δ/σ)_max_ < 0.001
1067 reflections	Δρ_max_ = 0.14 e Å^−^^3^
112 parameters	Δρ_min_ = −0.19 e Å^−^^3^
1 restraint	Extinction correction: *SHELXTL*'(Sheldrick, 2008), Fc^*^=kFc[1+0.001xFc^2^λ^3^/sin(2θ)]^-1/4^
Primary atom site location: structure-invariant direct methods	Extinction coefficient: 0.0108 (8)

### Special details

**Table:**

Geometry. All e.s.d.'s (except the e.s.d. in the dihedral angle between two l.s. planes) are estimated using the full covariance matrix. The cell e.s.d.'s are taken into account individually in the estimation of e.s.d.'s in distances, angles and torsion angles; correlations between e.s.d.'s in cell parameters are only used when they are defined by crystal symmetry. An approximate (isotropic) treatment of cell e.s.d.'s is used for estimating e.s.d.'s involving l.s. planes.
Refinement. Refinement of *F*^2^ against ALL reflections. The weighted *R*-factor *wR* and goodness of fit *S* are based on *F*^2^, conventional *R*-factors *R* are based on *F*, with *F* set to zero for negative *F*^2^. The threshold expression of *F*^2^ > σ(*F*^2^) is used only for calculating *R*-factors(gt) *etc*. and is not relevant to the choice of reflections for refinement. *R*-factors based on *F*^2^ are statistically about twice as large as those based on *F*, and *R*- factors based on ALL data will be even larger.

### Fractional atomic coordinates and isotropic or equivalent isotropic displacement parameters (Å^2^)

**Table:**

	*x*	*y*	*z*	*U*_iso_*/*U*_eq_	
O1	0.02008 (12)	0.12376 (4)	0.3335 (3)	0.0530 (5)	
N1	−0.08464 (14)	0.07777 (6)	0.2532 (4)	0.0530 (6)	
H1A	−0.1185	0.0952	0.1991	0.064*	
H1B	−0.1013	0.0530	0.2556	0.064*	
N2	0.15555 (13)	0.04420 (5)	0.1786 (2)	0.0364 (5)	
N3	0.12698 (13)	0.00771 (5)	0.1195 (3)	0.0413 (5)	
C1	−0.00707 (15)	0.08880 (6)	0.3326 (3)	0.0380 (5)	
C2	0.04624 (16)	0.05620 (6)	0.4265 (3)	0.0435 (6)	
H2A	0.0163	0.0307	0.4032	0.052*	
H2B	0.0441	0.0609	0.5551	0.052*	
C3	0.14666 (15)	0.05349 (7)	0.3680 (3)	0.0391 (5)	
H3B	0.1771	0.0788	0.3924	0.047*	
H3A	0.1775	0.0329	0.4379	0.047*	
C4	0.2226 (2)	0.10885 (7)	0.0702 (5)	0.0622 (8)	
H4B	0.2746	0.1082	0.1504	0.093*	
H4C	0.1747	0.1253	0.1210	0.093*	
H4A	0.2414	0.1198	−0.0434	0.093*	
C5	0.18687 (15)	0.06742 (6)	0.0435 (3)	0.0422 (5)	
C6	0.17798 (19)	0.04516 (8)	−0.1109 (4)	0.0505 (6)	
H6A	0.1938	0.0529	−0.2270	0.061*	
C7	0.14038 (16)	0.00856 (7)	−0.0582 (3)	0.0438 (6)	
C8	0.1147 (2)	−0.02660 (9)	−0.1705 (5)	0.0628 (8)	
H8A	0.1465	−0.0499	−0.1274	0.094*	
H8B	0.1319	−0.0217	−0.2932	0.094*	
H8C	0.0491	−0.0309	−0.1634	0.094*	

### Atomic displacement parameters (Å^2^)

**Table:**

	*U*^11^	*U*^22^	*U*^33^	*U*^12^	*U*^13^	*U*^23^
O1	0.0548 (10)	0.0323 (7)	0.0719 (13)	−0.0018 (6)	−0.0196 (10)	−0.0002 (8)
N1	0.0488 (10)	0.0381 (9)	0.0720 (16)	−0.0038 (8)	−0.0163 (12)	0.0039 (11)
N2	0.0386 (10)	0.0338 (10)	0.0369 (10)	0.0008 (7)	−0.0009 (9)	−0.0020 (8)
N3	0.0410 (10)	0.0342 (9)	0.0486 (12)	0.0007 (7)	−0.0011 (10)	−0.0057 (8)
C1	0.0391 (11)	0.0335 (9)	0.0415 (12)	0.0025 (8)	0.0025 (10)	−0.0037 (9)
C2	0.0473 (13)	0.0385 (11)	0.0447 (13)	0.0024 (9)	0.0045 (11)	0.0049 (10)
C3	0.0419 (12)	0.0367 (10)	0.0387 (12)	0.0061 (9)	−0.0027 (11)	−0.0005 (9)
C4	0.0787 (19)	0.0442 (13)	0.0636 (18)	−0.0134 (12)	0.0057 (17)	0.0057 (13)
C5	0.0425 (11)	0.0419 (10)	0.0422 (13)	−0.0005 (9)	0.0004 (12)	0.0018 (10)
C6	0.0521 (14)	0.0604 (16)	0.0390 (12)	−0.0001 (11)	0.0008 (12)	0.0009 (11)
C7	0.0365 (11)	0.0509 (14)	0.0441 (13)	0.0051 (9)	−0.0047 (11)	−0.0113 (11)
C8	0.0568 (15)	0.0671 (16)	0.0645 (19)	0.0018 (12)	−0.0065 (15)	−0.0243 (15)

### Geometric parameters (Å, °)

**Table:**

O1—C1	1.232 (2)	C3—H3A	0.9700
N1—C1	1.320 (3)	C4—C5	1.490 (3)
N1—H1A	0.8598	C4—H4B	0.9600
N1—H1B	0.8603	C4—H4C	0.9600
N2—C5	1.347 (3)	C4—H4A	0.9600
N2—N3	1.359 (3)	C5—C6	1.373 (4)
N2—C3	1.448 (3)	C6—C7	1.394 (4)
N3—C7	1.335 (3)	C6—H6A	0.9300
C1—C2	1.505 (3)	C7—C8	1.488 (4)
C2—C3	1.518 (3)	C8—H8A	0.9600
C2—H2A	0.9700	C8—H8B	0.9600
C2—H2B	0.9700	C8—H8C	0.9600
C3—H3B	0.9700		
			
C1—N1—H1A	120.2	C5—C4—H4B	109.5
C1—N1—H1B	119.8	C5—C4—H4C	109.5
H1A—N1—H1B	120.0	H4B—C4—H4C	109.5
C5—N2—N3	112.13 (18)	C5—C4—H4A	109.5
C5—N2—C3	129.18 (18)	H4B—C4—H4A	109.5
N3—N2—C3	118.66 (18)	H4C—C4—H4A	109.5
C7—N3—N2	104.88 (19)	N2—C5—C6	106.28 (19)
O1—C1—N1	122.5 (2)	N2—C5—C4	123.4 (2)
O1—C1—C2	121.3 (2)	C6—C5—C4	130.3 (2)
N1—C1—C2	116.14 (18)	C5—C6—C7	106.0 (2)
C1—C2—C3	113.56 (19)	C5—C6—H6A	127.0
C1—C2—H2A	108.9	C7—C6—H6A	127.0
C3—C2—H2A	108.9	N3—C7—C6	110.7 (2)
C1—C2—H2B	108.9	N3—C7—C8	120.2 (2)
C3—C2—H2B	108.9	C6—C7—C8	129.1 (3)
H2A—C2—H2B	107.7	C7—C8—H8A	109.5
N2—C3—C2	112.11 (19)	C7—C8—H8B	109.5
N2—C3—H3B	109.2	H8A—C8—H8B	109.5
C2—C3—H3B	109.2	C7—C8—H8C	109.5
N2—C3—H3A	109.2	H8A—C8—H8C	109.5
C2—C3—H3A	109.2	H8B—C8—H8C	109.5
H3B—C3—H3A	107.9		

### Hydrogen-bond geometry (Å, °)

**Table:**

*D*—H···*A*	*D*—H	H···*A*	*D*···*A*	*D*—H···*A*
N1—H1A···O1^i^	0.86	2.10	2.936 (3)	164
N1—H1B···N3^ii^	0.86	2.30	3.084 (3)	152
C3—H3B···O1^iii^	0.97	2.52	3.413 (3)	154

Symmetry codes: (i) *x*−1/4, −*y*+1/4, *z*−1/4; (ii) −*x*, −*y*, *z*; (iii) *x*+1/4, −*y*+1/4, *z*+1/4.
